# Structure of amyloid β_25–35_ in lipid environment and cholesterol-dependent membrane pore formation

**DOI:** 10.1038/s41598-019-38749-7

**Published:** 2019-02-25

**Authors:** Nabin Kandel, Jason O. Matos, Suren A. Tatulian

**Affiliations:** 1 Physics Graduate Program, University of Central Florida, Orlando, FL USA; 2 Biotechnology Graduate Program, University of Central Florida, Orlando, FL USA; 3 Department of Physics, University of Central Florida, Orlando, FL USA; 4 Present Address: Graduate Program in Biochemistry and Biophysics, Brandeis University, Waltham, MA USA

## Abstract

The amyloid β (Aβ) peptide and its shorter variants, including a highly cytotoxic Aβ_25–35_ peptide, exert their neurotoxic effect during Alzheimer’s disease by various mechanisms, including cellular membrane permeabilization. The intrinsic polymorphism of Aβ has prevented the identification of the molecular basis of Aβ pore formation by direct structural methods, and computational studies have led to highly divergent pore models. Here, we have employed a set of biophysical techniques to directly monitor Ca^2+^-transporting Aβ_25–35_ pores in lipid membranes, to quantitatively characterize pore formation, and to identify the key structural features of the pore. Moreover, the effect of membrane cholesterol on pore formation and the structure of Aβ_25–35_ has been elucidated. The data suggest that the membrane-embedded peptide forms 6- or 8-stranded β-barrel like structures. The 8-stranded barrels may conduct Ca^2+^ ions through an inner cavity, whereas the tightly packed 6-stranded barrels need to assemble into supramolecular structures to form a central pore. Cholesterol affects Aβ_25–35_ pore formation by a dual mechanism, i.e., by direct interaction with the peptide and by affecting membrane structure. Collectively, our data illuminate the molecular basis of Aβ membrane pore formation, which should advance both basic and clinical research on Alzheimer’s disease and membrane-associated pathologies in general.

## Introduction

Proteolytic cleavage of the amyloid precursor protein (APP) produces the amyloid β (Aβ) peptide, which forms extracellular fibrillar deposits in cross β-sheet conformation^1–3^. Identification of amyloid plaques in the brains of Alzheimer’s patients led to the amyloid cascade hypothesis, directly relating Aβ deposits with the etiology of the disease^4–6^. Further evidence identified the prefibrillar soluble oligomers of Aβ as the most cytotoxic entities causing neuronal cell dysfunction and death^7–10^.

Aβ peptide occurs in brain tissue in various forms. The 40- and 42-amino acid residue peptides, Aβ_1–40_ and Aβ_1–42_, are the dominant species, with the latter being less abundant but more toxic^8^. Shorter variants of Aβ, resulting from truncation by various proteases, are also found in human brain. Among these, the 11amino acid residue Aβ_25–35_ peptide (GSNKGAIIGLM) is highly cytotoxic and has been the subject of extensive research on the mechanism of action of Aβ and modulation of its toxicity^11–15^. There is strong evidence that the 25–35 segment of Aβ plays an important role in the aggregation properties and cytotoxicity of the peptide^11,16,17^. Moreover, similar cytotoxic effects of Aβ_25–35_ and Aβ_1–42_, involving DNA damage, transcription dysregulation, and apoptosis, have been reported^18–20^, suggesting these peptides may share common mechanism of toxicity.

Aβ_25–35_ cytotoxicity has been shown to involve mitochondrial membrane permeabilization through activation of expression of mitochondrial permeability transition pore protein(s)^21,22^. In addition, studies on direct Aβ_25–35_-membrane interactions suggested membrane binding, insertion, and ion-conducting pore formation by the peptide^17,23–29^. Aβ_25–35_ binds to anionic membranes, promoted by its excess positive charge due to Lys_28_, as well as to zwitterionic phosphatidylcholine (PC) membranes, although less efficiently^28–30^. Inhibition of membrane binding reduced the peptide’s toxic effect^24^, providing additional support for the membranotropic mechanism of Aβ_25–35_ action.

Despite the evidence for membrane permeabilization by Aβ_25–35_, identification of membrane pores and their structural characterization has not been achieved. Two populations of membrane-bound peptide have been described for both artificial and cellular membranes, peripheral and membrane-inserted^29,31–35^, and the latter population is thought to form the pathogenic, ion conducting pores. Molecular dynamics (MD) simulations produced an 8-stranded β-barrel-like model for Aβ_25–35_, with 3.5 to 4.0Å inner diameter^36^, similar to “cylindrins” proposed by Do *et al*.^37^. Other MD studies offered a different, α-helical model of membrane pores formed by Aβ_25–35_, i.e., a bundle of eight α-helical peptides and 16 cholesterol molecules, with a large, 14.6Å wide cavity^26,27,38^.

Cholesterol binding to Aβ may play a role in the peptide’s toxic effect, especially considering the elevated levels of cholesterol and its oxidized forms during Alzheimer’s disease and promotion of Aβ aggregation and cytotoxicity by cholesterol^39,40^. Cholesterol binding to a region of APP that encompasses the Aβ_25–35_ sequence has been identified by magnetic resonance techniques^41^. The side chain amide of Asn_27_ was proposed to be H-bonded to the hydroxyl of cholesterol, whereas Gly_29_, Ile_32_, and Gly_33_ were involved in nonpolar interactions with the sterol backbone^41^. Molecular modeling studies suggested that in case of membrane-inserted Aβ_25–35_, but not for the longer Aβ_1–40_ and Aβ_22–35_ peptides, the side chain amine of Lys_28_ forms H-bonding with cholesterol hydroxyl group^27^.

Different forms of Aβ have been shown to preferentially interact with cholesterol as compared to PC, a major lipid in cellular membranes^26,27,38,42,43^, which may explain the promotion of Aβ neurotoxicity by cholesterol and inhibition by PC^12,44,45^. On the contrary, MD data implied that competitive interaction of cholesterol with Aβ_1–42_ prevents its aggregation and β-sheet formation, thereby protecting the membranes from peptide-induced disruption^46,47^. Furthermore, cholesterol was shown to suppress Aβ_25–35_ peptide’s toxicity by hindering formation of pathogenic aggregates^48^. Dual effect of cholesterol, i.e. assisting membrane insertion of Aβ_25–35_ at low cholesterol content via direct interaction with the peptide and expelling the peptide from the membrane at high cholesterol content through a membrane stiffening effect, has also been reported^49^.

The structure of membrane pores formed by Aβ_25–35_ remains unknown, as structurally diverse β-sheet and α-helical models have been predicted by MD methods^26,36^, and the effect of cholesterol on the structure of the membrane-bound peptide and its function awaits elucidation. In this work, biophysical methods have been employed to monitor and characterize Ca^2+^-transporting pore formation by Aβ_25–35_, to determine the structure of the peptide in lipid environment, and to identify the effect of cholesterol on both the structure and function of the pores. The data lead to 6- or 8-stranded β-barrel like pore structures, which conduct ions either directly, through an inner opening of the barrel, or by assembling into supramolecular structures, with the pore between tightly packed barrels. Cholesterol exerts its effect on pore formation by two competing mechanisms, i.e., by affecting the membrane structure and direct interaction with the peptide. Together, our data elucidate the molecular mechanism of membrane pore formation by the smallest and most toxic fragments of Aβ and hence contribute to the understanding of Aβ cytotoxicity in general.

## Results and Discussion

### Membrane pore formation

**Figure 1:**
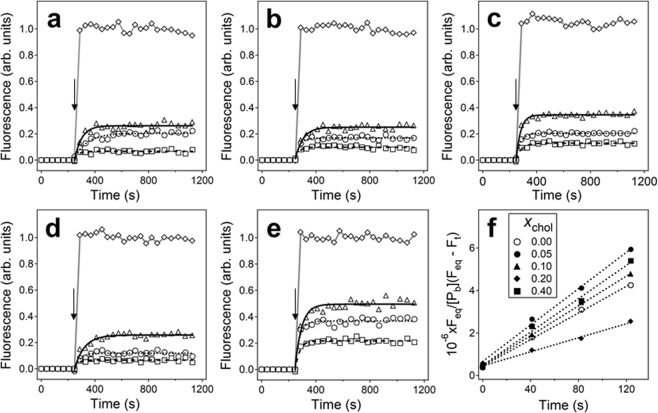
(**a**–**e**) Time dependence of fluorescence of Quin-2 entrapped in lipid vesicles in a buffer of 75 mM NaCl, 30 mM myo-inositol, 6 mM CaCl_2_, 20 mM Tris-HCl (pH 7.2). Aβ_25–35_ was added at 248 s (indicated by downward arrows) at peptide-to-lipid molar ratios of 1:10 (squares), 1:5 (circles), or 1:3 (triangles). Vesicle membranes contained 0.3 mol fraction of POPG and varying contents of POPC and cholesterol. The mol fraction of cholesterol was *x*_chol_ = 0.00, 0.05, 0.10, 0.20, and 0.40 (panels a–e, respectively) and the mol fraction of POPC was *x*_POPC_ = 0.7 − *x*_chol_. Total lipid concentration was 200 μM. Data of peptide-induced rise in fluorescence are fitted with single exponential lines, using rate constants shown in Fig. 2b. Data presented by rhombs are obtained upon addition of non-fluorescent Ca^2+^ ionophore 4-Br-A23187. In all cases, the maximum effect induced by 4-Br-A23187 is normalized to 1.0. Panel (f) presents the relative increase of fluorescence during the first two minutes following peptide addition, at defined mol fractions of membrane cholesterol, as indicated in the inset. The slopes of these linear plots were used to determine the second order rate constants of pore formation, *k*_a_.

Quin-2 loaded unilamellar lipid vesicles were prepared in a way that the internal buffer contained 6 mM Quin-2 and no CaCl_2_, while the external buffer contained 6 mM CaCl_2_ and no Quin-2. Sequestration of Quin-2 from Ca^2+^ ions resulted in weak Quin-2 fluorescence. Addition of a non-fluorescent Ca^2+^ ionophore 4-Br-A23187 to the vesicles resulted in rapid increase of Quin-2 fluorescence to *F*_max_, whereas addition of the peptide resulted in an exponential increase of fluorescence, which gradually reached an equilibrium level, *F*_eq_, indicating membrane permeabilization and interaction between Quin-2 and Ca^2+^ ions (Fig. 1a–e). Single-exponential fitting of peptide-induced change of fluorescence produced two parameters, i.e., *F*_rel_ = *F*_eq_/*F*_max_ and the experimentally observed rate constant, *k*_exp_. These parameters have been determined for membranes containing 0.3 mol fraction of an anionic lipid 1-palmitoyl-2-oleoyl-*sn*-glycero-3-phosphoglycerol (POPG), (0.7 − *x*_chol_) mol fraction of a zwitterionic lipid 1-palmitoyl-2-oleoyl-*sn*-glycero-3-phosphocholine (POPC), and *x*_chol_ mole fraction of cholesterol, with *x*_chol_ = 0, 0.05, 0.10, 0.20, and 0.40, at three peptide concentrations for each *x*_chol_.

**Figure 2:**
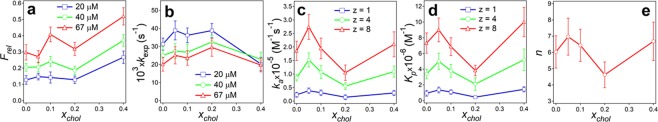
Effect of membrane cholesterol on the relative equilibrium Quin-2 fluorescence (**a**), single exponential rate constant of increase in Quin-2 fluorescence following peptide addition (**b**), second-order rate constant of pore formation (**c**), affinity constant of peptide oligomers within the membrane (**d**), and the number of oligomers in the pore structure. (**e**) In panels (a,b), data for 3 peptide concentrations are presented, as indicated. In panels (c,d), data for each *z* (number of monomers in a pre-formed oligomer) have been averaged for all 3 peptide concentrations. Panel (e) shows mean values of *n*, averaged for 3 peptide concentrations, with standard deviations. Average values and standard deviations have been generated from three independent experiments. Total lipid concentration was 200 μM.

If all vesicles contained peptide-induced Ca^2+^-transporting pores, then *F*_eq_ would eventually reach the level of fluorescence induced by the ionophore, *F*_max_, corresponding to *F*_rel_ = 1.0. However, data of Figs 1 and 2a show that values of *F*_eq_ increase with peptide concentration but do not reach *F*_max_. This effect has been observed earlier^28,50^ and explained by a conjecture that although all vesicles have plenty of bound peptide oligomers, only a small number of these oligomers form fully functional pore structures, resulting in a fraction of vesicles lacking Ca^2+^-transporting pores. Incomplete targeting of the vesicles or transient effects of the peptide could also account for partial vesicle permeabilization, as described earlier for antimicrobial peptides^51–53^. This explanation is consistent with the positive correlation between *F*_rel_ and the peptide concentration (Fig. 2a) and implies that Aβ_25–35_ oligomers assemble within the membrane into a heterogeneous set of structures, possibly reflecting the inherent polymorphism of Aβ peptide and its fragments^54–56^.

Earlier studies showed that, upon incubation in aqueous buffer, the Aβ_25–35_ peptide forms oligomers of up to eight monomers, which transition from unordered conformation to mostly β-sheet/β-turn structure^28^. These oligomeric structures stabilized at 2–3 hours of incubation, and the absence of fibrils was documented by negligible Thioflavin-T fluorescence during this time period. In addition, vesicle leakage experiments showed that the peptide sample incubated for ~2.5 h had the highest pore forming capability. Therefore, the peptide was stirred in buffer for 2.5 h before adding to the vesicles. Membrane binding of the peptide and subsequent pore formation are proposed to proceed as follows. The oligomers contain *z* monomers, with *z* varying from 1 to 8, as shown by microelectrophoresis studeis^28^. Following addition to lipid vesicles, peptide monomers or oligomers bind to membranes and associate with each other within the membrane to form transmembrane pores composed of *n* units. The second-order rate constants of this process, *k*_a_, have been evaluated from the linearized kinetics of Quin-2 fluorescence increase and the concentration of membrane-bound peptide particles, [*P*_b_] (see Eq. 2 in Methods). The values of [*P*_b_] at each bulk concentration of the peptide have been determined previously for vesicles containing 10 mol % cholesterol and buffers of various ionic strengths^28^. The slopes of the linear plots of *F*_eq_/{[*P*_b_](*F*_eq_ − *F*_t_)} vs. time (Fig. 1f), along with the values of [*P*_b_] corresponding to the ionic conditions of this study, were used to quantitate *k*_a_ at varying peptide concentrations and *x*_chol_ (Fig. 2c). Furthermore, the experimentally measured single-exponential rate constants, *k*_exp_, and the second-order rate constants, *k*_a_, allowed evaluation of the intermolecular affinity constants of the peptide within the membrane, *K*_p_, through Eq. 3 (Fig. 2d). Finally, the number of peptide oligomers in the pore, *n*, was estimated through Eq. S1 of Supplementary Information, making use of *K*_p_ and [*P*_b_] (Fig. 2e).

Parameters *k*_a_ and *K*_p_ have been evaluated for three different oligomeric states of Aβ_25–35_ peptide, *z* = 1, 4 and 8, as prior studies have suggested that on incubation in buffer for 2.5 hours, the peptide forms assemblies varying from monomers to octamers^28^. The affinity between peptide octamers reaches 10^7^ M^−1^, and the second order rate constant exceeds 2.5×10^5^ M^−1^s^−1^, whereas the corresponding values for the monomers are smaller by an order of magnitude (Fig. 2c,d). Both *k*_a_ and *K*_p_ initially increase at *x*_chol_ = 0.05, drop with further increase of *x*_chol_ to 0.2, and rise again at *x*_chol_ = 0.4 (Fig. 2c,d). The average number of oligomers in the pore structure, *n*, displays a similar dependence on membrane cholesterol content; *n* is in the range 6 to 8 at *x*_chol_ = 0.0–0.1 and *x*_chol_ = 0.4 but acquires lower values of around 5 at *x*_chol_ = 0.2 (Fig. 2e). Correlation of *k*_a_ and *K*_p_ with the number of peptide oligomers in the pore structure indicates more efficient pore formation capability by larger peptide assemblies.

Association of externally added Ca^2+^ ions with intravesicular Quin-2 could result from rupture of the vesicles rather than membrane pore formation, which should be accompanied with decrease in light scattering by the sample. To check this possibility, right angle static light scattering was measured at the end of kinetic measurements of Quin-2 fluorescence. Data of Fig. S1 of Supplementary Information show that light scattering increases upon addition of the peptide, and this effect is proportional to the peptide concentration. This result suggests that a) the vesicles maintain their integrity in the presence of Aβ_25–35_ and b) the peptide apparently causes vesicles aggregation, which may result from reduction of electrostatic repulsion between anionic vesicles upon binding of the cationic peptide.

### Peptide structure assessed by circular dichroism

**Figure 3:**
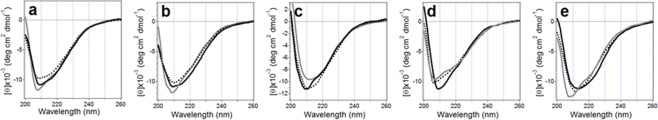
Circular dichroism spectra of Aβ_25–35_ peptide in the presence of unilamellar vesicles composed of POPG, POPC, and cholesterol at mol fractions of 0.3, (0.7 − *x*_chol_), and *x*_chol_, where *x*_cho_ = 0.00, 0.05, 0.10, 0.20, 0.40 (a-e, respectively). Total lipid concentration was 200 μM. Gray, black dotted, and black solid lines correspond to peptide-to-lipid molar ratios of 1:10, 1:5, and 1:3, respectively. The buffer was the same as in Fig. 1.

Circular dichroism (CD) spectra were recorded after each Quin-2 kinetics measurements to assess the structure of the Aβ_25–35_ peptide in the presence of lipid vesicles. At peptide-to-lipid molar ratio of 1:3 and *x*_chol_ = 0 to 0.2, the spectra displayed a minimum at 208 nm and a shoulder around 220 nm, indicative of a secondary structure dominated by type I β-turn, with minimal fractions of β-sheet and α-helix^57–59^ (Fig. 3a–d). At *x*_chol_ = 0.4, the α-helix fraction increased at the expense of β-sheet and β-turn (Fig. 3e). At peptide-to-lipid molar ratios 1:5 and 1:10, the α-helical feature at 220 nm diminished, suggesting predominantly β-sheet/β-turn structure of the peptide.

At equilibrium, a fraction of the total peptide pool is membrane-bound and the rest is free in the buffer. CD spectra of the Aβ_25–35_ peptide free in aqueous buffer, as well as the fractions of membrane-bound peptide (*f*_bound_) at varying ionic strengths of the buffer have been evaluated earlier^28^. This information has been used here to simulate CD spectra of the membrane-bound peptide by subtracting the spectra of the peptide in the buffer (Fig. S2a of Supplementary Information), multiplied by (1 − *f*_bound_), from the spectra measured in the presence of vesicles (Fig. 3). The difference spectra displayed a deep minimum at 221–223 nm, assigned to the backbone *n*-π* transition, and a weaker minimum around 280 nm, generated by the aromatic side chains (Fig. S2b). An α-helical peptide in aqueous medium would produce two minima at 208 and 222 nm, and a typical spectrum of a β-sheet would display a minimum around 216 nm^57,59^. The spectra shown in Fig S2b do not match any of these patterns. Recalling that the *n*-π* transition is very sensitive to the polarity of the environment and undergoes significant red-shift in nonpolar media^59,60^, and that for β-sheet structures the position of the *n*-π* band can be shifted by several nanometers depending on structural details^61^, the signal at 221–223 nm may be attributed to a membrane-inserted β-sheet that is red-shifted by 6 nm due to the hydrophobic membrane core. Still the interpretation of this outcome, which is based on spectral manipulation, may not be sufficiently substantiated. Therefore, the secondary structure of the Aβ_25–35_ peptide inserted in lipid membranes has been determined directly using ATR-FTIR spectroscopy, as described below.

### Effect of cholesterol on membrane fluidity

In order to identify the effect of cholesterol on the membrane structural dynamics, Laurdan fluorescence spectroscopy was used. Laurdan is a lipophilic fluorophore, which upon excitation (350–366 nm) generates emission spectrum that is sensitive to the viscosity of the local environment. In a rigid environment, the emission maximum occurs at 435 nm, and in a more fluid-like environment the emission shifts to 500 nm^62^. In the absence of cholesterol, Laurdan exhibited a two-component emission spectrum, with nearly equal intensities at 435 and 500 nm, whereas addition of cholesterol up to 0.4 mol fraction resulted in gradual decline of the 500 nm component and increase in the 435 nm component, suggesting membrane solidification (Fig. S3 of Supplementary Information).

In the absence of cholesterol, the POPC/POPG membrane is in liquid-disordered (L_d_) phase, and increasing cholesterol content causes transition into a more rigid, liquid-ordered (L_o_) phase^63^. More rigid membranes that contain a larger fraction of cholesterol may resist membrane insertion of Aβ_25–35_ and pore formation. However, data of Figs 1 and 2c,d indicate a more complex, non-monotonous effect of cholesterol on pore formation by the peptide, which can be interpreted in terms of an interplay between two effects, i.e., interaction of cholesterol with the peptide and modulation of the membrane structure by cholesterol. At *x*_chol_ = 0.05, cholesterol supports pore formation, as noted by an increase in *k*_a_ and *K*_p_ (Fig. 2c,d), presumably by direct interaction with the peptide^26,27,42,43^. Notably, under this favorable conditions the pore structure contains 8 oligomers, on average (Fig. 2e), and in light of our earlier studies, each of these oligomers contains up to 8 peptide monomers^28^. Increase of *x*_chol_ to 0.2 results in a reduction of the number of oligomers in the pore to around 5 with a concomitant decrease of the pore formation parameters *k*_a_ and *K*_p_ (Fig. 2c–e). Considering that at cholesterol contents exceeding 20–25 mol % the L_o_ phase becomes dominant^63^, this effect may result from squeezing out of larger assemblies of the peptide. At a higher cholesterol content of *x*_chol_ = 0.40, the membrane-condensing effect of cholesterol saturates and the disordered boundaries between phospholipid-rich and cholesterol-rich domains become dominant^64^, which may promote peptide insertion and pore formation, as evidenced by the increase in both *k*_a_ and *K*_p_ (Fig. 2c,d).

### Peptide structure from ATR-FTIR spectroscopy

The structure of Aβ_25–35_ peptide totally embedded in lipid bilayers was studied using attenuated total reflection Fourier transform infrared (ATR-FTIR) spectroscopy. Lipid multilayers, containing Aβ_25–35_ at 1:15 peptide-to-lipid molar ratio, were deposited on a germanium plate and spectra were measured at parallel (||) and perpendicular (⊥) polarizations of the infrared light relative to the plane of incidence (see Methods). Following recordings of the spectra of the dry sample, similar measurements were performed for the lipid-peptide system that was nominally hydrated (humidified) by D_2_O vapor and then totally hydrated by bulk D_2_O-based buffer.

**Figure 4:**
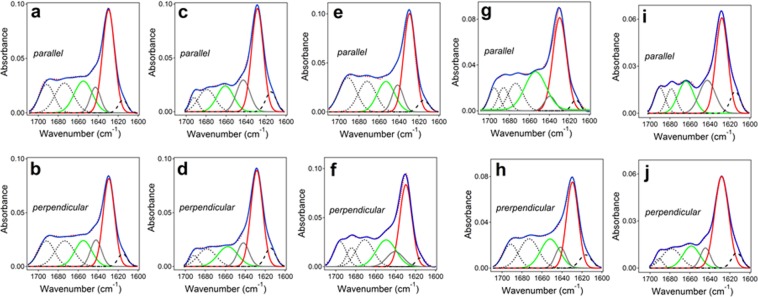
ATR-FTIR spectra of Aβ_25–35_ peptide in lipid multilayers composed of POPG, POPC, and cholesterol at mol fractions of 0.3, (0.7 − *x*_chol_), and *x*_chol_, respectively, where *x*_chol_ = 0.00 (**a**,**b**), 0.05 (**c**,**d**), 0.10 (**e**,**f**), 0.20 (**g**,**h**), and 0.40 (**i**,**j**). Spectra were measured at || and ⊥ polarizations, as indicated by labels “parallel” and “perpendicular.” The lipid-peptide system was exposure to D_2_O-based buffer containing 50 mM NaCl and 50 mM Na,K-phosphate in D_2_O (pH* = 6.8). The measured spectra, shown in red dotted lines, were curve-fitted to generate the amide I components that are shown under the spectra as follows: turns–black dotted lines, α-helix–green solid lines, irregular structure–gray solid lines, β-sheet–red solid lines, and side chains–black dashed lines. The curvefit, i.e. the sum of all components, is shown for each spectrum in solid blue line.

The ATR-FTIR spectra of the dry lipid-peptide samples in the amide I region display a major component at 1630-1628 cm^−1^, assigned to β-sheet structure, and additional components of lower intensities assigned to α-helix (1660-1654 cm^−1^), irregular (unordered) structure (1644-1642 cm^−1^), and β- and/or γ-turns (1700-1670 cm^−1^)^65,66^ (Fig. S4 of Supplementary Information). The lower frequency components (1620-1600 cm^−1^) are assigned to the side chains. Hydration with D_2_O vapor or bulk D_2_O-based buffer caused appreciable changes in the spectra, yet the β-sheet remained the dominant structure (Fig. S5 of Supplementary Information and Fig. 4).

**Figure 5:**
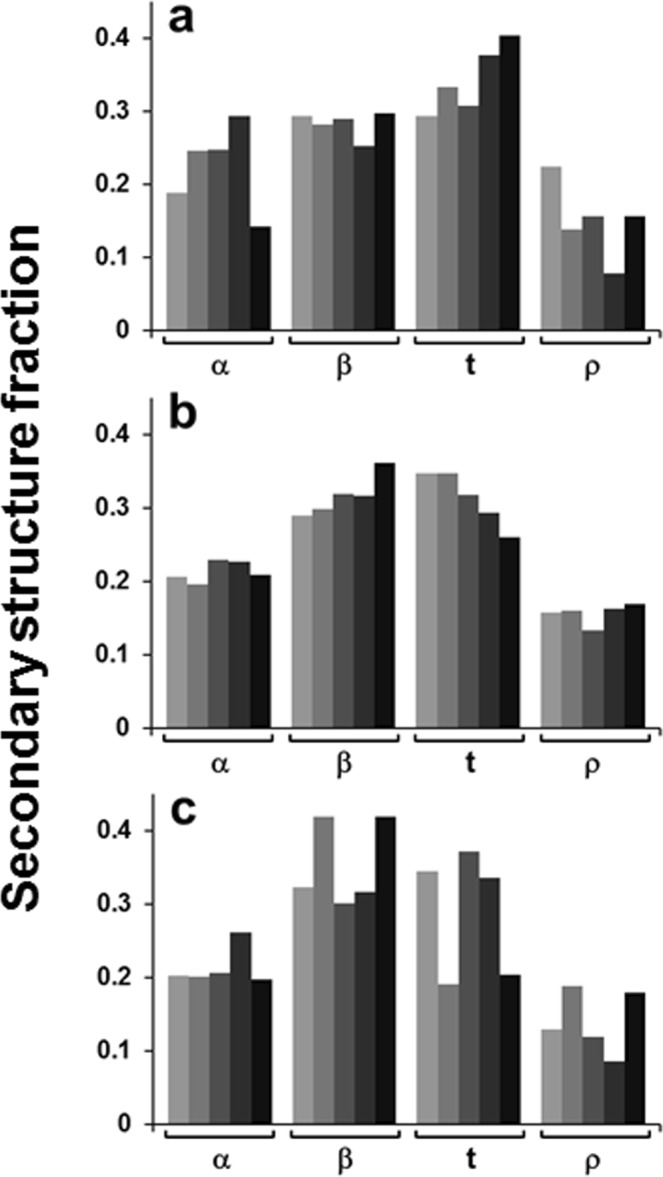
Secondary structure of the Aβ_25–35_ peptide in lipid multilayers composed of POPG, POPC, and cholesterol at mol fractions of 0.3, (0.7 − *x*_chol_), and *x*_chol_, respectively, in dry state (**a**), humidified by D_2_O vapor (**b**), and exposed to D_2_O-based buffer containing 50 mM NaCl and 50 mM Na,K-phosphate, pH* = 6.8. (**c**) In each panel, α-helix (α), β-sheet (β), turn (t), and irregular (ρ) structure fractions are presented for membranes with varying cholesterol contents, i.e., *x*_chol_ = 0.00, 0.05, 0.10, 0.20, and 0.40; increasing darkness of the bars corresponds to increasing *x*_chol_.

The relative areas of amide I components were used to evaluate the fractions of secondary structure of the Aβ_25–35_ peptide in membranes in dry state, hydrated from gas phase by D_2_O vapor, or exposed to bulk buffer, as shown in Fig. 5. In dry state, the content of β-sheet structure is just below 30% and that of β-turns is 30–40%, and the latter increases with increasing cholesterol content in the membrane (Fig. 5a). The fraction of α-helix is around 20%, and the irregular structure constitutes a small fraction of ~10%. If all peptide molecules were in a nearly identical conformation, then these fractions would imply approximately 3–4 amino acid residues in each of β-sheet and β-turn structures and 1–2 residues for both α-helix and irregular structure. In this scenario, the presence of α-helix could be disregarded because one α-helical turn requires at least 4 residues. Such interpretation may not reflect the reality, however, because Aβ peptide and its fragments are known to be inherently polymorphic^54–56^. An alternative, more plausible interpretation is that a significant peptide population, up to 40–50%, assumes mostly β-sheet structure and the rest is in β-turn and α-helix conformation, with a few unordered amino acids between them.

Upon hydration with D_2_O vapor, the β-sheet fraction increases and β-turn fraction decreases with increasing cholesterol content (Fig. 5b). Under conditions that can be compared more directly with the vesicle experiments, i.e. in the presence of bulk aqueous buffer, the β-sheet structure reaches the maximum fraction of 40% at *x*_chol_ = 0.05 and 0.40, while β-turns decrease to around 20% at these cholesterol contents (Fig. 5c). Notably, the vesicle permeabilization potency of the peptide is maximal at *x*_chol_ = 0.05 and 0.40, as judged from the dependence of *k*_a_ and *K*_p_ on *x*_chol_ (Fig. 2c,d). This result suggests that the functional membrane pores formed by Aβ_25–35_ are in β-sheet conformation.

### Peptide orientation from ATR-FTIR spectroscopy

Further insight into the structure of membrane pores was gained by evaluating the orientation of the peptide molecules in the membrane. Above data indicated that the peptide assumes β-sheet conformation upon pore formation. Hence, the pore can be modeled as a β-barrel-like structure. The ATR dichroic ratios of the β-sheet components of ATR-FTIR spectra were determined as *R*_β_ = *a*_β,_||/*a*_β,⊥_, where *a*_β,_|| and *a*_β,⊥_ are the areas of β-sheet components of amide I bands measured at || and ⊥ polarizations, respectively. The orientation of β-strands with respect to the barrel central axis (angle β) was determined through Eq. S6 for the angle γ between the membrane normal and barrel axis varying within a conceivable range, from 0 to 20 degrees. Data summarized in Table S1 of Supplementary Information indicate that, for γ = 0°, the angle between β-strands and the barrel axis varies between 20 and 27 degrees with a few exceptions when it acquires smaller values. When γ = 20° is used, smaller values for the angle β are obtained. For membranes under aqueous buffer at *x*_chol_ = 0.4, values of γ greater that 10° cannot be combined with the measured β-sheet dichroic ratio *R*_β_ = 0.89 to solve Eq. S6 for angle β. This means that data analysis using γ = 0° is more reliable, or the β-barrel-like oligomeric structures are tilted from the membrane normal by less than 10°. It should be noted, however, that the tilt angles presented in Table S1 are the ensemble average values, implying there is a normal angular distribution around these values.

### Lipid order from ATR-FTIR spectroscopy

**Figure 6:**
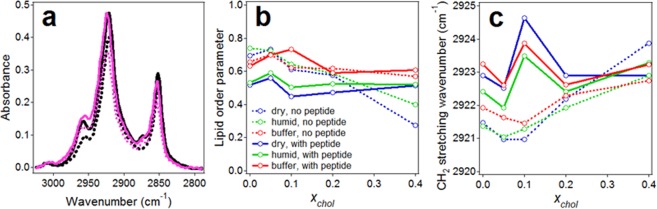
(**a**) Lipid acyl chain methylene stretching vibrational spectra in the absence (black) and presence (magenta) of Aβ_25–35_ peptide at 1:15 peptide/lipid molar ratio at parallel (solid lines) and perpendicular (dotted lines) polarizations of the incident light. The lipid layers contained 60 mol % POPC, 30 mol % POPG, and 10 mol % cholesterol and were humidified by D_2_O vapor. (**b**) Lipid acyl chain order parameter as a function of molar fraction of cholesterol (*x*_chol_) in the absence and presence of Aβ_25–35_ peptide at 1:15 peptide/lipid molar ratio under defined conditions, as indicated. (**c**) Lipid acyl chain methylene antisymmetric stretching peak wavenumbers as a function of molar fraction of cholesterol in the absence and presence of Aβ_25–35_ peptide; line assignments in (**c**) are the same as in (**b**).

To check the quality of lipid multilayers deposited on germanium plate and the effect of the Aβ_25–35_ peptide on the membrane, lipid acyl chain order parameters and CH_2_ vibrational frequencies were analyzed using polarized ATR-FTIR spectra in the methylene stretching region. Representative spectra, shown in Fig. 6a, display two major absorbance peaks at 2923-2921 cm^−1^ and 2853-2851 cm^−1^ generated by antisymmetric and symmetric CH_2_ stretching modes, respectively^65,66^. Lipid dichroic ratios were determined as *R*_L_ = *a*_L,_||/*a*_L,⊥_, where *a*_L,_|| and *a*_L,⊥_ are the total areas of lipid CH_2_ stretching bands measured at || and ⊥ polarizations, respectively. The acyl chain order parameters, *S*_L_, were evaluated through Eq. S4, using angle α = 90° for the CH_2_ stretching transition dipole moment^66^. As seen from Eq. S5, the order parameter can vary between extreme values of −0.5 and 1.0, corresponding to orientation of the molecular axis (in this case, the lipid acyl chain) perpendicular and along the membrane normal, respectively. A value of *S* = 0 indicates no preferred orientation, i.e., disorder. Another measure of lipid order is the peak vibrational wavenumber, which increases by 4–5 cm^−1^ upon lipid phase transition from solid to fluid phase^65^.

Data of Fig. 6b show that, in the absence of cholesterol, the values of lipid order parameter vary between 0.5 and 0.7. Considering the presence of a double bond in the *sn*-2 acyl chains of both POPC and POPG, which tends to tilt the chain by adopting *cis*-conformation, these values indicate well-ordered membranes. For comparison, *S*_L_ for POPC/POPG multilayers was in the range 0.3 to 0.6^67^, and *S*_L_ for monolayers of dipalmitoylphosphatidylcholine in solid state, with all-*trans* acyl chains, was around 0.7^66^. The order parameter of POPC in extruded vesicles varied between 0.24 and 0.80, depending on protein binding^68^, again indicating that the lipid order in supported multilayers under buffer is similar to that in vesicles floating in buffer. In the absence of the peptide, *S*_L_ slightly increases at *x*_chol_ = 0.05, then decreases, especially at *x*_chol_ = 0.4 (Fig. 6b, dotted lines), implying membrane ordering effect of cholesterol at low *x*_chol_ and a disordering effect at high *x*_chol_. Consistent with this trend, the CH_2_ stretching wavenumbers decrease at *x*_chol_ = 0.05–0.1 and increase at higher cholesterol content (Fig. 6c, dotted lines). The presence of the peptide tends to reduce the lipid order parameter and increase the CH_2_ stretching wavenumbers at low *x*_chol_, implying a membrane disordering effect (Fig. 6b,c). Interestingly, at higher cholesterol contents the peptide’s lipid disordering effect is suppressed, reminiscent of the conclusions from recent MD studies^69^. It seems plausible that the peptide accumulates cholesterol around itself, leaving the lipid phase with a low fraction of cholesterol, which exerts its ordering effect. Also, in the presence of the peptide, *S*_L_ considerably increases upon hydration with buffer.

Together, these data provide evidence that the germanium-deposited lipid multilayers with the membrane-embedded peptide are very well ordered, especially in fully hydrated state, mimicking the vesicles in aqueous buffer. Therefore, the structure and the orientation of the Aβ_25–35_ peptide deduced from ATR-FTIR experiments can be extrapolated to the membrane pore structure in unilamellar vesicles.

### Pore structure

Above data show the Aβ_25–35_ peptide forms Ca^2+^ transporting pores in lipid membranes, where it adopts mostly β-sheet conformation. Our earlier data suggested the peptide forms small oligomers (up to octamers) during pre-incubation in buffer, and these oligomers assemble into the pore structure in the membrane^28^. Data of Fig. 2e indicate the functional pore may contain 5 to 8 such peptide units. Our polarized ATR-FTIR data provide experimental evidence for a β-barrel-like model of Aβ_25–35_ pores and show that the β-strands are tilted by 22±4 degrees from the barrel axis.

**Figure 7:**
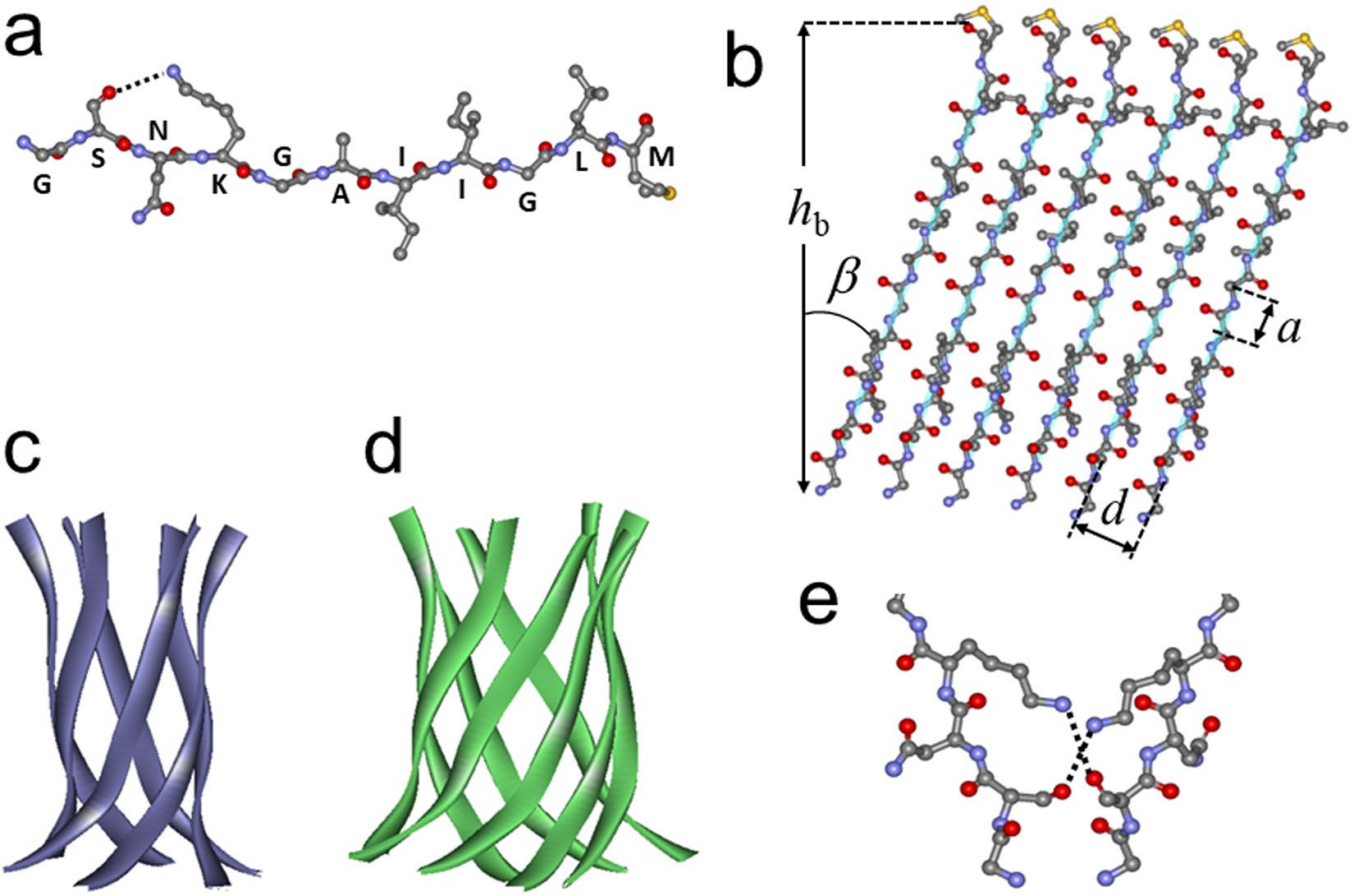
(**a**) Model for Aβ_25–35_ peptide structure is shown as a coiled and twisted β-strand in ball and stick format, with H-bonding between the hydroxyl oxygen of Ser_26_ side chain and the amino group of Lys_28_ side chain (dotted line). Atoms are colored as follows: carbons gray, nitrogens blue, oxygens red, sulfur yellow. Hydrogens are omitted for simplicity. Single-letter amino acid labels are placed at respective C_α_ atoms. (**b**) Model for an unwrapped 6-stranded β-barrel of Aβ_25–35_ peptide, viewed from inside the barrel. Ball and stick details are as in panel (a), and each backbone is traced by a turquoise line ribbon. Upon coiling into a cylindrical structure, the first and last strands will be associated through H-bonding. (**c**,**d**) Cartoons for a 6- and 8-stranded β-barrel-like structures of Aβ_25–35_ peptide in ribbon format, constructed using the structure of a11-amino acid long segment (residues 90–100) of αB crystallin (PDB entry 3sgn), which forms 6-stranded β-barrel-like “cylindrin” structure^86^. (**e**) Proposed H-bonding between side chains of Ser_26_ and Lys_28_ of a strand in one β-barrel with Lys_28_ and Ser_26_ of a strand in an adjacent β-barrel, stabilizing the supramolecular pore structure.

These constraints, together with established geometric parameters of β-barrels, allow identification of critical details of the pore structure. The side chains of consecutive amino acids in a β-strand are oriented alternately in and out of the barrel, and the H-bonding direction between the strands is nearly perpendicular to the side chains and strand axes^70–73^. Figure 7a shows a β-strand formed by the Aβ_25–35_ peptide, with the side chains in the plane and the interstrand H-bonding normal to the plane of the page. We consider two structures: 1) Ser_26_, Lys_28_, Ala_30_, Ile_32_, Leu_34_ are inside the barrel and Gly_25_, Asn_27_, Gly_29_, Ile_31_, Gly_33_, Met_35_ are outside and 2) vice versa. Using the van der Waals volumes of amino acids (48 Å^3^ for Gly, 67 Å^3^ for Ala, 73 Å^3^ for Ser, 96 Å^3^ for Asn, 124 Å^3^ for Ile, Leu, Met, and 135 Å^3^ for Lys), the total volume required to accommodate the inward oriented side chains of six strands is calculated to be 3138 Å^3^ and 2928 Å^3^ for topologies 1 and 2, respectively. For an 8-stranded barrel, the respective volumes are 4184 Å^3^ and 3904 Å^3^.

For a barrel composed of *z* strands tilted relative to the cylindrical axis by an angle β, the barrel radius is^70^:

1 $$R=\frac{d}{2\,\sin (\frac{\pi }{z})\cos \,\beta }$$

where *d* is the interstrand distance (see Fig. 7b). For standard β-barrels, *d* = 4.72Å and the displacement along the strand axis per amino acid residue is *a* = 3.48 Å^70,74^. The internal volume of the barrel is *V* = π*R*^2^*h*_b_, where *h*_b_ is the barrel height: *h*_b_ = *ma*cosβ, *m* being the number of amino acids in the strand (in our case, *m* = 11). Using β = 22°, for 6- and 8-stranded barrels we obtain *R*_6_ = 5.09Å, *V*_6_ = 2890 Å^3^, and *R*_8_ = 6.65Å, *V*_8_ = 4930Å^3^. Thus, the inner volume of a 6-stranded barrel may accommodate the inward side chains of strands in topology 2. For example, if angle β = 24°, which is within the measured range for upright oriented barrels (γ = 0°, Table S1), the interior of a 6-stranded barrel provides 2933 Å^3^ space, perfect for side chain packing of strands in topology 2. A 6-stranded barrel in topology 1 may be possible but it will require deviation from standard barrel geometry. An 8-stranded barrel will easily accommodate the side chains in either configuration, with extra free volume of 750–1000 Å^3^. While tight packing of inward oriented side chains may stabilize the barrel structure, both 6- and 8-stranded structures are possible, as schematically shown in Fig. 7c,d.

**Figure 8:**
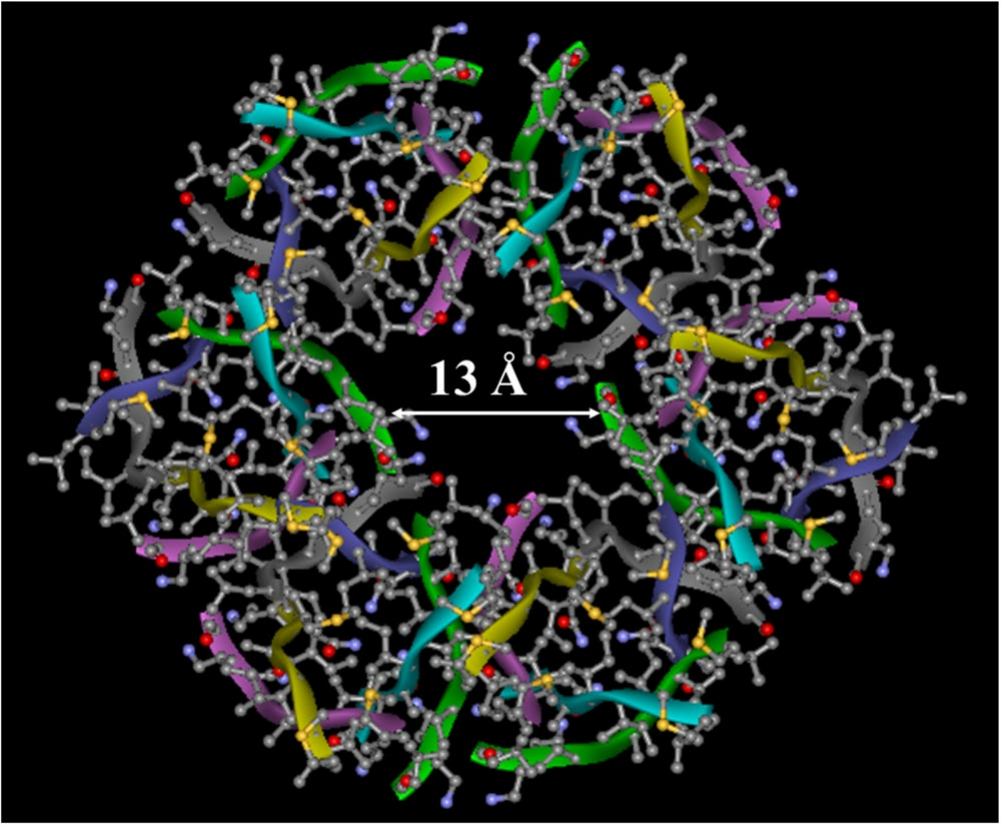
Model for the membrane pore formed by the Aβ_25–35_ peptide, as viewed along the pore central axis (top view). The pore complex is a hexamer of 6-stranded β-barrel-like structures. The barrel structure is constructed based on the backbone structure of αB crystallin residues 90–100 (PDB entry 3sgn)^86^, side chains replaced with those of Aβ_25–35_. Each barrel is turned about its cylindrical axes by 60 degrees relative to its neighbors. The diameter of the central ion-conducting cavity is around 13Å, as shown.

Our data suggest that 6-stranded barrel-like structures are tightly packed. Eight-stranded structures, which have been identified as the largest Aβ_25–35_ assemblies formed in aqueous buffer before addition to vesicles^28^, with 750–1000 Å^3^ cavities might ideally provide a channel 2.6–3.0 Å in radius (given *h*_b_ = 11 × 3.48 × cos22° = 35.5Å). However, the free volume is more likely to be randomly distributed without providing a well-defined passageway. The possible candidates for Ca^2+^-transporting structures thus appear to be supramolecular structures made of five to eight (Fig. 2e) 6-stranded barrels or, less likely, 8-stranded barrels. In the former case, interactions between the barrels may involve Asn-Asn H-bonding, Ile-Ile hydrophobic interactions, or possibly glycine zipper-type contacts between Asn-Gly-Ile-Gly motifs in topology 1. In case of topology 2 configuration, interstrand Lys-Ser H-bonding, facilitated by interaxial twist of interacting strands (Fig. 7e), and Ile-Leu hydrophobic interactions are possible. Considering *R*_6_ = 5.09Å (see above) and allowing a ~2Å thick layer for the side chains, a hexamer of 6-stranded barrels would provide a pore of 6–7Å radius (Fig. 8). These pores may acquire cation selectivity due to the terminal carbonyl oxygens not involved in H-bonding because of interstrand staggering.

Considering the significant energy required for Ca^2+^ dehydration (>10 kcal/mol)^75^, the ions likely pass through the channel in hydrated state. The pore opening formed by such structures is sufficiently wide, 6–7Å in radius, to transport hydrated Ca^2+^ ions, as documented in this work. Eight-stranded pores are possible, but may not be able to efficiently conduct ions through the opening in a single barrel-like structure.

Pore-like structures of various Aβ peptides, including Aβ_1–42_, Aβ_1–40_, Aβ_9–42_, Aβ_17–42_, and Aβ_25–35_, have been described earlier^26,27,36,76–84^. The structural features of these ion-conducting pore models varied in a wide range; from single octameric β-barrel^36^ or α-helical structures^26,27^ to supramolecular assemblies composed of multiple barrel-like structures, such as hexamers of 6-stranded β-barrels of Aβ_1–42_^81^, and tetramers or pentamers of 16-stranded β-barrels of Aβ_9–42_ and Aβ_17–42_^82^. Protofibrillar oligomers of Aβ_1–42_, which caused significant ionic currents in lipid bilayers at ≥2μM concentrations, were modeled as annular structures composed of around six oligomeric units^79^. The pore model proposed in this work is a hexamer of 6-stranded structures (Fig. 8), similar to these annular structures as well as those proposed by Shafrir *et al*. for Aβ_1–42_^81^.

In summary, our data shed light on the elusive membrane pores formed by the Aβ_25–35_ peptide, which were modeled earlier by computational methods either as β-barrel-like structures or an α-helical bundle^26,27,36^. Not only transmembrane Ca^2+^ transport through Aβ_25–35_ pores is documented, but also the kinetic parameters and the stoichiometry of pore formation and the key structural features of the pore are identified, which is a significant step forward in understanding the molecular basis of Aβ cytotoxicity through a membrane damaging mechanism.

## Methods

Detailed description of the methods used in this work is presented in the Supplementary Information. Briefly, the following procedures have been used.

To prepare unilamellar vesicles, chloroform solutions of POPC, POPG, and cholesterol were mixed at various molar proportions and the solvent was removed by desiccation. The dry lipid was suspended in an aqueous buffer containing 6 mM Quin-2 and extruded through 100 nm pore-size polycarbonate membranes. The vesicles were passed through a Sephadex G-50 column to remove external Quin-2, and 6 mM CaCl_2_ was added. Thus, intravesicular Quin-2 was sequestered from external Ca^2+^ ions.

Hexafluoroisopropanol (HFIP) solution of the synthetic Aβ_25–35_ peptide was dried by desiccation and suspended in an aqueous buffer by stirring for 2.5 h. A certain volume of Quin-2 loaded vesicles was placed in a quartz cuvette and the baseline fluorescence of Quin-2 was measured, using a J-810 spectropolarimeter with a fluorescence attachment. Addition of the peptide resulted in gradual increase in fluorescence, indicating Ca^2+^ influx and association with Quin-2. In positive control experiments, non-fluorescent Ca^2+^ ionophore 4-Br-A23187 was added, which produced the maximum increase in Quin-2 fluorescence. CD measurements were performed using the same J-810 instrument.

For ATR-FTIR experiments, the chloroform solution of POPG, POPC, and cholesterol was mixed with HFIP solution of Aβ_25–35_ at a 1:15 peptide/(total lipid) molar ratio and spread over a germanium plate, which served as an internal reflection element. The sample was dried by desiccation, the plate was assembled in an ATR cell and placed in a Vector 22 FTIR spectrometer. Spectra were collected at || and ⊥ polarizations. The cell was disassembled and the lipid-peptide sample was exposed to D_2_O vapors, followed by collection of FTIR spectra at || and ⊥ polarizations. A D_2_O-based buffer was then injected into the cell and spectra were measured again at both polarizations.

Most theoretical procedures used in data analysis are described in the Supplementary Information. The theory of pore formation kinetics has been presented earlier^85^ and is briefly described here. Upon addition of the peptide to the lipid vesicles, the peptide, in monomeric or oligomeric form, binds to the membrane and assembles to form Ca^2+^-permeable pores by stepwise association with one another. Formation of a functional pore is followed by Ca^2+^ influx and binding to intravesicular Quin-2, resulting in gradual increase in Quin-2 fluorescence. The pore formation is considered a second-order process that can be described by the following relationship:

2 $$\frac{{F}_{eq}}{[{P}_{b}]({F}_{eq}-{F}_{t})}=\frac{1}{[{P}_{b}]}+{k}_{a}t$$

where *F*_t_ is Quin-2 fluorescence intensity at time *t* after peptide addition, *F*_*eq*_ is the saturation level of fluorescence, [*P*_b_] is the concentration of membrane-bound peptide, and *k*_*a*_ is the second-order rate constant in units M^−1^s^−1^. Values of [*P*_b_] at various total peptide concentrations have been determined earlier, by microelectrophoresis^28^. Values of *F*_*eq*_/{[*P*_b_](*F*_*eq*_ − *F*_*t*_)} were determined from Quin-2 fluorescence measurements at defined time points and plotted against time. The slopes of these linear plots (Fig. 1f) allowed evaluation of the second-order rate constants of pore formation, *k*_*a*_. It has been shown that peptide-peptide association within the membrane is a dynamic, reversible process, and the formation of the stable, functional structure is the rate limiting step of pore formation^28,85^. In this case, the values of *k*_*a*_ and the experimentally observed rate constants of Quin-2 fluorescence increase, *k*_*exp*_, can be used to determine the peptide-peptide affinity constant within the membrane, *K*_*p*_:

3 $${K}_{p}=\frac{{k}_{a}}{{k}_{\exp }}$$

## Supplementary information

Supplementary Information
