# Supplementary material for: Structure of amyloid β25–35 in lipid environment and cholesterol-dependent membrane pore formation
Source: Sci Rep. 2019 Feb 25;9:2689. doi: 10.1038/s41598-019-38749-7 (PMC6389947; doi:10.1038/s41598-019-38749-7)
Supplement: Supplementary file 1 — Supplementary Information [file 41598_2019_38749_MOESM1_ESM.pdf]

Supplementary Information

Structure of amyloid $\beta_{25\text{-}35}$ in lipid environment and cholesterol-dependent membrane pore formation

Nabin Kandel[1,†], Jason O. Matos[2,†,‡], and Suren A. Tatulian[3]*

[1] Physics Graduate Program, University of Central Florida, Orlando, FL, USA
[2] Biotechnology Graduate Program, University of Central Florida, Orlando, FL, USA
[3] Department of Physics, University of Central Florida, Orlando, FL, USA
[†] These authors contributed equally to this work
[‡] Current address: Graduate Program in Biochemistry and Biophysics, Brandeis University, Waltham, MA, USA
* Correspondence should be addressed to S.A.T. (Email: statulia@ucf.edu; Fax: 407-823-5112; Tel: 407-823-1543)

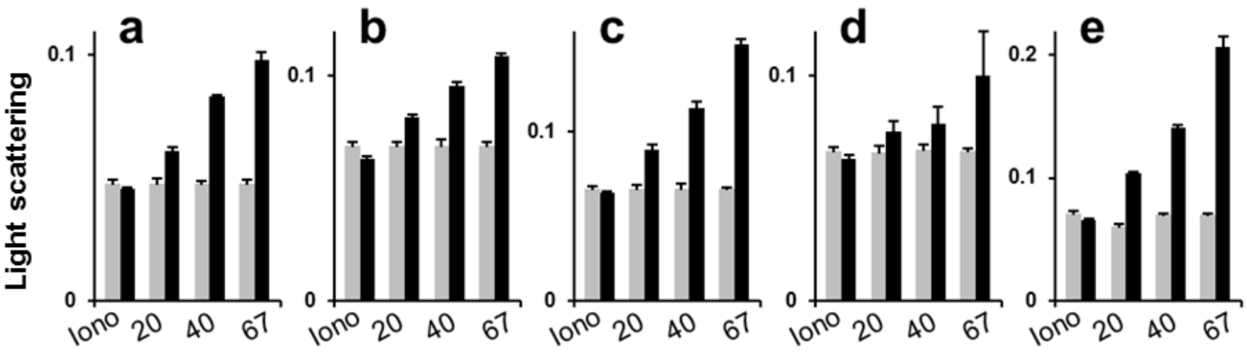

Figure S1. Light scattering at right angle from lipid vesicles (arbitrary units), recorded after kinetic measurements of Quin-2 fluorescence. Vesicles were composed of POPG, POPC, and cholesterol at mol fractions of 0.3, $(0.7 - x_{\text{chol}})$, and x_{chol}, respectively, where $x_{\text{chol}} = 0.00$ (a), 0.05 (b), 0.10 (c), 0.20 (d), and 0.40 (e). Gray and black bars correspond to light scattering before and after addition of the ionophore (Iono) or the $A\beta_{25\text{-}35}$ peptide at indicated peptide concentrations, in μM. Average values and standard deviations from 5 measurements are shown. The incident wavelength was 550 nm. The buffer was composed of 75 mM NaCl, 30 mM myo-inositol, 6 mM $CaCl_2$, 20 mM Tris-HCl (pH 7.2).

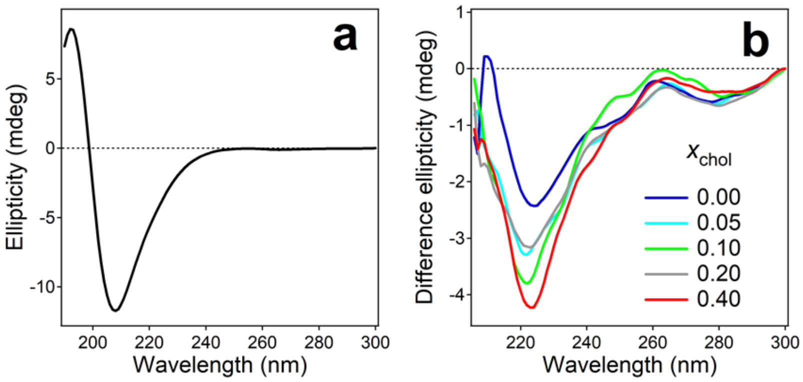

Figure S2. Circular dichroism spectrum of Aβ$_{25\text{-}35}$ peptide in aqueous buffer without vesicles (a) and simulated spectra of the peptide bound to lipid vesicles (b). The spectra of membrane-bound peptide shown in panel (b) have been simulated by subtracting the spectrum of free peptide in aqueous buffer (a), multiplied by a scaling factor, from the spectra of the peptide in the presence of lipid vesicles composed of POPG, POPC, and cholesterol at mol fractions of 0.3, (0.7 – x_{chol}), and x_{chol}, as indicated.

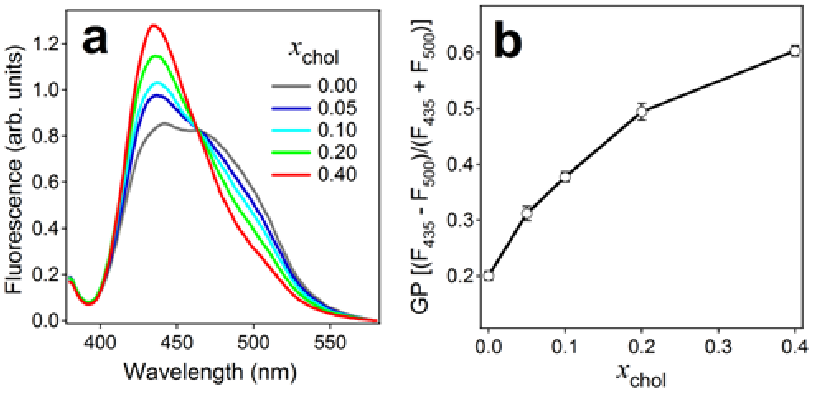

Figure S3. Dependence of membrane fluidity on cholesterol content. (a): Laurdan fluorescence spectra of lipid vesicles composed of 0.3 mol fraction of POPG, (0.7 – x_{chol}) mol fraction of POPC, and x_{chol} mol fraction of cholesterol, as indicated. Excitation wavelength was at 360 nm. (b): Generalized polarization (GP) of Laurdan as a function of mol fraction of cholesterol in vesicle membranes, averaged from four experiments, with the total lipid concentration varying from 100 μM to 400 μM. The buffer was the same as in Fig. S1.

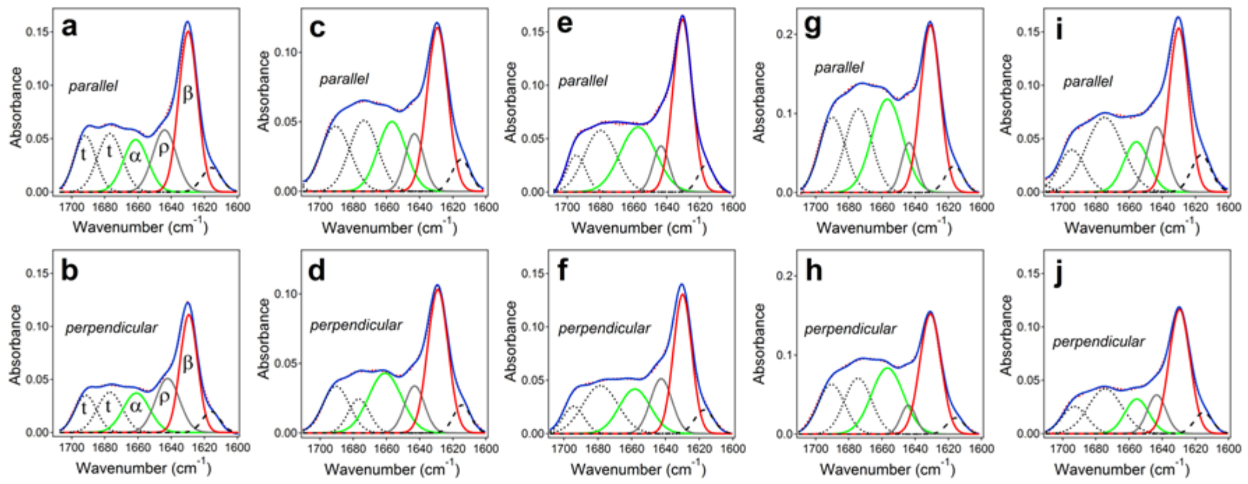

Figure S4. ATR-FTIR spectra of dry samples of Aβ$_{25\text{-}35}$ peptide in lipid multilayers. The lipid composition was POPG, POPC, and cholesterol at mol fractions of 0.3, (0.7 – x_{chol}), and x_{chol}, respectively, where x_{chol} = 0.00 (a,b), 0.05 (c,d), 0.10 (e,f), 0.20 (g,h), and 0.40 (i,j). At each x_{chol}, spectra were measured at ∥ and ⊥ polarizations, as indicated by labels "parallel" and "perpendicular." The measured spectra, shown in red dotted lines, were curve-fitted to generate the amide I components that are shown under the spectra as follows: turns–black dotted lines, α-helix–green solid lines, irregular structure–gray solid lines, β-sheet–red solid lines, and side chains–black dashed lines. In panels (a) and (b), turn, α-helix, irregular structure, and β-sheet are labeled t, α, ρ, and β, respectively. The curvefit, i.e. the sum of all components, is shown for each spectrum in solid blue line.

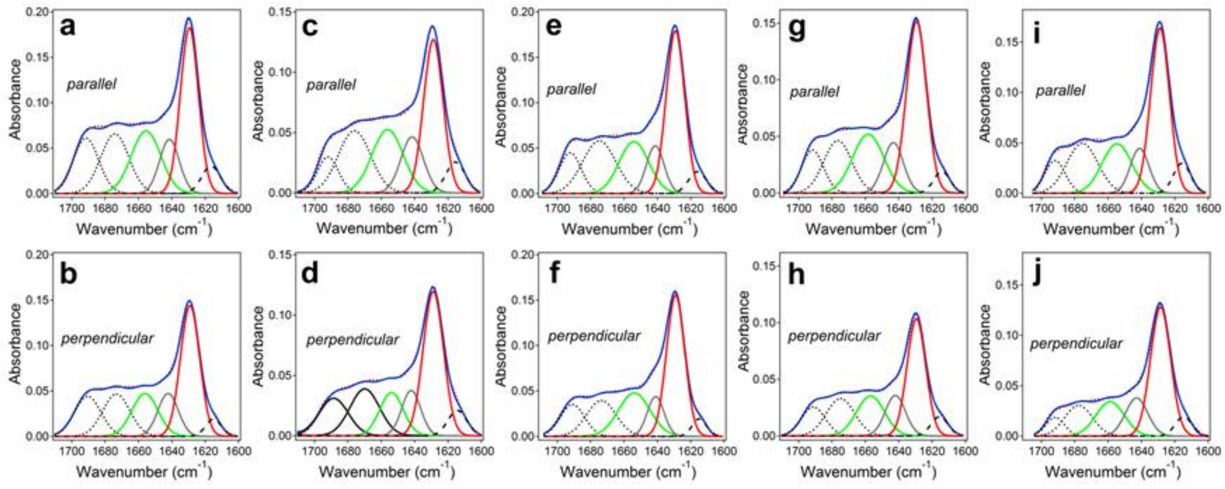

Figure S5. ATR-FTIR spectra of Aβ$_{25\text{-}35}$ peptide in lipid multilayers humidified by exposure to D_2O vapor. The lipid composition was POPG, POPC, and cholesterol at mol fractions of 0.3, (0.7 – x_{chol}), and x_{chol}, respectively, where x_{chol} = 0.00 (a,b), 0.05 (c,d), 0.10 (e,f), 0.20 (g,h), and 0.40 (i,j). Spectra were measured at ∥ and ⊥ polarizations, as indicated by labels "parallel" and "perpendicular." All other details are as in Fig. S4.

x_{chol}	Dry			D$_2$O vapor			D$_2$O buffer		
	R_β	β (°)		R_β	β (°)		R_β	β (°)	
		γ = 0°	γ = 20°		γ = 0°	γ = 20°		γ = 0°	γ = 20°
0.00	1.464	27.2	25.3	1.200	21.2	17.2	1.233	22.1	18.5
0.05	1.165	20.1	15.6	1.010	14.6	4.6	1.066	16.9	10.3
0.10	1.328	24.5	21.8	1.163	20.2	15.7	1.160	20.1	15.6
0.20	1.303	23.9	20.9	1.577	29.3	28.0	1.243	22.4	18.9
0.40	1.247	22.5	19.1	1.194	21.1	17.0	0.890	7.1	*

Table S1. Orientation of β-strands of Aβ$_{25\text{-}35}$ peptide in lipid multilayers under varying conditions.
*For R_β = 0.89, Eq. S6 excludes values of γ greater than 10 degrees.

Materials and Methods

Materials

The synthetic Aβ$_{25\text{–}35}$ peptide, acetylated at N-terminus and amidated at C-terminus, was purchased from Peptide 2.0 Inc. (Chantilly, VA, USA) and was ≥98% pure, as verified by high performance liquid chromatography and mass-spectrometry. Quin-2 tetrapotassium salt was from EMD Chemicals (San Diego, CA). Hexafluoroisopropanol (HFIP), non-fluorescent Ca^{2+} ionophore 4-Br-A23187, salts, buffers, and most of other reagents were purchased from Sigma-Aldrich (St. Louis, MO, USA) or Fisher Scientific (Hanover Park, IL, USA). The synthetic lipids, 1-palmitoyl-2-oleoyl-*sn*-glycero-3-phosphocholine (POPC), 1-palmitoyl-2-oleoyl-*sn*-glycero-3-phosphoglycerol (POPG), and ovine wool cholesterol, were from Avanti Polar Lipids (Alabaster, AL, USA) and were of ≥98% purity. The fluorescent lipid 6-dodecanoyl-*N*,*N*-dimethyl-2-naphthylamine (Laurdan) was from Sigma-Aldrich (St. Louis, MO).

Experimental procedures

Membrane permeabilization experiments. Chloroform solutions of POPG (an anionic lipid), POPC (a zwitterionic lipid), and cholesterol were mixed at molar proportions of 0.3/(0.7 – x_{chol})/x_{chol}, where x_{chol} is the molar fraction of cholesterol and was 0.00, 0.05, 0.10, 0.20, or 0.40. The solvent was removed by desiccation, the dry lipid mixture was suspended in an aqueous buffer containing 75 mM KCl, 6 mM Quin-2 tetrapotassium salt, 20 mM Tris-HCl (pH 7.2), and extruded through 100 nm pore-size polycarbonate filters using a mini extruder (Avanti Polar Lipids). The lipid suspension was passed through a column loaded with Sephadex G-50 resin, equilibrated with 75 mM NaCl, 30 mM myo-inositol, 20 mM Tris-HCl (pH 7.2), to remove external Quin-2. Elution samples containing the highest lipid content, assessed by apparent turbidity, were collected and the total lipid concentration was adjusted to 0.2 mM based on a calibration curve, i.e. dependence of right-angle static light scattering on lipid concentration. Adjustment of the lipid concentration was paralleled with addition of 6 mM $CaCl_2$ to the vesicles. Care was taken to maintain the osmotic balance, using myo-inositol when necessary. Thus, intravesicular Quin-2 was sequestered from external Ca^{2+} ions.

For membrane permeabilization experiments, a certain amount of HFIP solution of the Aβ$_{25-35}$ peptide was dried by desiccation, then aqueous buffer (75 mM NaCl, 20 mM Tris-HCl, pH 7.2) was added and the sample was stirred in a glass vial for 2.5 hours. To monitor peptide-induced membrane permeabilization, a certain volume of Quin-2 loaded vesicles was placed in a quartz cuvette with 4 mm × 4 mm internal cross section and the baseline fluorescence of Quin-2, entrapped in lipid vesicles, was measured at excitation wavelength of 339 nm and emission wavelengths between 450 and 600 nm, using a J-810 spectropolarimeter with a fluorescence attachment (Jasco, Tokyo, Japan). Upon establishment of a stable baseline, a certain dose of the peptide, pre-incubated in aqueous buffer as described above, was added and consecutive fluorescence spectra were collected for 15 minutes until the increase in fluorescence approached saturation. In positive control experiments, non-fluorescent Ca^{2+} ionophore 4-Br-A23187 was added, which produced the maximum increase in Quin-2 fluorescence, F_{max}. In negative control experiments, blank buffer was added, which only resulted in slight decrease in fluorescence due to sample dilution. The rate constant of the exponential increase in Quin-2 fluorescence and the level of saturation were used to analyze the pore formation kinetics, as described in section Methods of the main text.

Circular dichroism and light scattering. At the end of fluorescence measurements, circular dichroism (CD) and right-angle static light scattering spectra of the vesicle samples were measured, using the same J-810 spectropolarimeter, to estimate the secondary structure of the peptide in the presence of vesicles and to verify the integrity of the vesicles. CD was measured between 180 and 320 nm. The spectra were normalized to obtain the mean residue molar ellipticity, $[\theta] = \theta_{\mathrm{meas}}/cn_{\mathrm{res}}l$, where θ_{meas} is the measured ellipticity in millidegrees, c is the molar concentration of the peptide, n_{res} is the number of amino acid residues in the peptide, and l is the optical path-length in millimeters. Light scattering was measured in the fluorescence mode, i.e., using the fluorescence photomultiplier mounted at 90 degrees relative to the incident beam, using incident wavelength at 550 nm and monitoring the intensity of scattered light between 535 and 565 nm. The signal intensity was obtained by subtracting the baseline value from the maximum.

Membrane fluidity. Membrane fluidity was measured by the method of Laurdan generalized polarization (GP). Laurdan was incorporated in vesicle membranes at 1 mol % and fluorescence spectra were measured between 380 and 580 nm with excitation at 360 nm. GP of Laurdan was evaluated as $\mathrm{GP} = (F_{435} - F_{500})/(F_{435} + F_{500})$, where F is the fluorescence emission intensity at respective wavelength.

Polarized ATR-FTIR experiments. Lipid multilayers with incorporated Aβ$_{25-35}$ peptide were prepared as follows. HFIP solution of the peptide was mixed with chloroform solutions of POPG, POPC, and cholesterol at molar proportions of POPG/POPC/cholesterol = 0.3/(0.7 – x_{chol})/x_{chol} and a peptide/(total lipid) molar ratio of 1:15. As in membrane leakage experiment, lipid layers with the following cholesterol fractions were studied: x_{chol} = 0.00, 0.05, 0.10, 0.20, and 0.40. The lipid-peptide solution was carefully and uniformly spread over a germanium plate (5.0 cm × 2.0 cm × 0.1 cm, cut at the 2.0 cm sides at a 45° aperture angle), which served as an internal reflection element in attenuated total reflection Fourier transform infrared (ATR-FTIR) experiments. The sample was air-dried, followed by desiccation for 1 hour. The plate, with the dry peptide-lipid sample deposited on one side, was assembled into a perfusable sample holder, which was mounted on a four-mirror ATR system (Buck Scientific, East Norwalk, CT, USA)

and placed in a Vector 22 FTIR spectrometer (Bruker, Billerica, MA, USA), equipped with a liquid nitrogen-cooled Hg/Cd/Te detector and an aluminum-grid-on-KRS-5 polarizer (Specac, Newmarket, Suffolk, UK). The spectrometer was purged with dry air (passed through a silica-gel column) for 15 minutes, then transmission spectra were collected at 2 cm^{-1} nominal resolution at parallel and perpendicular ($\parallel$ and $\perp$) polarizations, i.e., with plane-polarized incident light when the electric vector of the radiation is oriented parallel or perpendicular to the plane of incidence, respectively. Following completion of the measurements of the spectra of dry sample, the cell was disassembled and the plate with the lipid-peptide sample was exposed to D_2O vapors by incubation with hot (~90 ^oC) D_2O in a closed glass chamber for 15 minutes. Appearance of dew on the internal surface of the chamber indicated saturation of the internal volume with D_2O vapor. The sample, hydrated by D_2O from gas phase, was assembled in the cell and FTIR spectra were recorded at $\parallel$ and $\perp$ polarizations. Finally, a buffer composed of 50 mM NaCl and 50 mM Na,K-phosphate in D_2O (pH* = 6.8) was injected into the cell and the spectra of the sample exposed to bulk buffer were measured again at both polarizations. (pH* is the pH-meter reading and corresponds to pD = pH* + 0.4 = 7.2)[1,2]. In separate experiments, the transmission spectra of the bare germanium plate and those of identical lipid multilayers without the peptide were measured at $\parallel$ and $\perp$ polarizations, which were used as reference for calculation of the absorbance spectra.

Data analysis procedures

The oligomeric state of the pore. The oligomeric state of the pore has been evaluated based on a formalism of reversible aggregation, described in detail by Garg et al.[3] and in earlier publications cited therein. Values of relative equilibrium levels of Quin-2 fluorescence, $F_{rel} = F_{eq}/F_{max}$, along with peptide-peptide affinity constants (K_p) and membrane-bound peptide concentrations ($[P_b]$), were used to quantitate the number of peptide oligomers involved in pore formation, n, based on the following relationship[3]:

$$F_{rel} = q^{n-1}(n - nq + q) \qquad \text{(S1)}$$

where

$$q = \frac{1}{\alpha + \sqrt{\alpha^2 - 1}}$$

$$\alpha = 1 + \frac{1}{4K_p[P_b]}$$

Peptide structure from ATR-FTIR spectroscopy. The secondary structure of the peptide embedded in lipid layers was determined by analysis of the ATR-FTIR amide I absorbance band. The second derivatives of ATR-FTIR spectra measured at $\parallel$ and $\perp$ polarizations were used to identify the number and the locations of amide I components. Curve-fitting procedures were then carried out using the GRAMS software, which produced the actual amide I components. These components were assigned to certain secondary structures based on the following spectral ranges for each secondary structure: various types of turns (1700-1665 cm^{-1}), α-helix (1664-1648 cm^{-1}), irregular structure (1647 and 1639 cm^{-1}), β-sheet (1638-1620 cm^{-1})[4]. The low frequency amide I

components (1619-1600 cm^{-1}) were assigned to side chains and were subtracted from the amide I bands in secondary structure assessment procedures. Thus, the areas of components assigned to defined secondary structures were obtained for ∥ and ⊥ polarizations of the incident light: $a_{t,\parallel}$, $a_{\alpha,\parallel}$, $a_{\rho,\parallel}$, $a_{\beta,\parallel}$, $a_{t,\perp}$, $a_{\alpha,\perp}$, $a_{\rho,\perp}$, and $a_{\beta,\perp}$, where the subscripts t, α, ρ and β stand for turn, α-helix, irregular, and β-sheet structures, respectively. The relative areas of amide I components measured at a certain polarization cannot be directly used to evaluate the fractions of respective secondary structures because absorbance intensities depend not only on the content but also on the orientation of each structure. To identify the fraction of each secondary structure, first the genuine (polarization-independent) relative amide I areas were determined:

$$a_i = \frac{a_{i,\mathrm{II}} + G a_{i,\perp}}{a_{Total,\mathrm{II}} + G a_{Total,\perp}} \tag{S2}$$

In Eq. S2, $a_{\mathrm{Total},\parallel}$ and $a_{\mathrm{Total},\perp}$ are the total amide I areas at ∥ and ⊥ polarizations, respectively, corrected by subtraction of the side chain component, and G is a dimensionless scaling factor[5]. Under our experimental conditions, when the sample thickness exceeds the decay length of the evanescent wave, G = 1.44. The fraction of each secondary structure, f_i, was determined using the relative areas of all structures, corrected for respective extinction coefficient, ε_i:

$$f_i = \frac{a_i}{\varepsilon_i\left(\frac{a_\alpha}{\varepsilon_\alpha} + \frac{a_\beta}{\varepsilon_\beta} + \frac{a_t}{\varepsilon_t} + \frac{a_\rho}{\varepsilon_\rho}\right)} \tag{S3}$$

The following extinction coefficients have been used: $\varepsilon_\alpha = 5.1\times10^7$ cm/mol, $\varepsilon_\beta = 7.0\times10^7$ cm/mol, $\varepsilon_t = 5.5\times10^7$ cm/mol, and $\varepsilon_\rho = 4.5\times10^7$ cm/mol[4].

Peptide orientation from ATR-FTIR spectroscopy. The ATR dichroic ratios for each secondary structure were calculated as $R_i = a_{i,\parallel}/a_{i,\perp}$. The orientational order parameter, S, of a structure that has a molecular axis, such as an α-helix or an acyl chain of a lipid molecule, is defined as[4]:

$$S = \frac{2B}{\left(3\langle cos^2\alpha\rangle - 1\right)\left(B - 3E_z^2\right)} \tag{S4}$$

In Eq. S4, α is the angle between the transition dipole moment and the molecular axis, $B = E_x^2 - RE_y^2 + E_z^2$, E_x, E_y, and E_z are the orthogonal components of the incident infrared radiation, R is the ATR dichroic ratio, and the angular bracket indicates average value. Under our experimental conditions (a lipid-peptide multilayer on a germanium plate, with refractive indices of 1.43 and 4.00, respectively), E_x = 1.399, E_y = 1.514, and E_z = 1.621. For an α-helix, the angle between the transition diploe moment of amide I vibrational mode and helical axis is α = 39±1degrees, and for a lipid acyl chain in all-trans conformation, the angle between CH_2 stretching vibrations and the chain axis is α = 90 degrees[4-6]. The order parameters for α-helices

and lipid acyl chains were evaluated by Eq. S4. The average angle θ between the membrane normal and the molecular axis can be determined through the following relationship:

$$S = \frac{1}{2}\left(3\left\langle\cos^2\theta\right\rangle - 1\right) \tag{S5}$$

The orientation of β-strands arranged in a structure that has a central rotational axis of symmetry, such as a β-barrel-like pore, can be evaluated using the following relationship[6]:

$$\frac{1}{2}\left(3\left\langle\sin^2\beta\right\rangle - 1\right) = \frac{2B}{\left(3\left\langle\cos^2\gamma\right\rangle - 1\right)\left(B - 3E_z^2\right)} \tag{S6}$$

where β is the angle of the strand axis relative to the central axis of the pore and γ is the angle between pore axis and membrane normal. The ATR dichroic ratios of β-sheet components of amide I bands can be used to determine the strand orientations relative to the pore axis by Eq. S6 provided the angle γ is known. If angle γ cannot be determined experimentally, the problem can be solved by using conceivable values for the pore orientation within the membrane. In this work, the angle β for strand orientation was evaluated using values for γ varying between 0° and 20°.

Supplementary References

1. Glasoe, P. K. & Long, F. A. Use of glass electrodes to measure acidities in deuterium oxide. *J. Phys. Chem.* **64,** 188–190 (1960).
2. Makhatadze, G. I., Clore, G. M. & Gronenborn, A. M. Solvent isotope effect and protein stability. *Nature Struct. Biol.* **2,** 852–855 (1995).
3. Garg, P., Nemec, K. N., Khaled, A. R. & Tatulian, S. A. Transmembrane pore formation by the carboxyl terminus of Bax protein. *Biochim. Biophys. Acta* **1828,** 732–742 (2013).
4. Tatulian, S. A. Structural characterization of membrane proteins and peptides by FTIR and ATR-FTIR spectroscopy. *Methods Mol. Biol.* **974,** 177–218 (2013).
5. Marsh, D. Quantitation of secondary structure in ATR infrared spectroscopy. *Biophys. J. 77,* 2630-2637 (1999).
6. Marsh, D. Infrared dichroism of twisted beta-sheet barrels. The structure of *E. coli* outer membrane proteins. *J. Mol. Biol.* **297,** 803–808 (2000).
